# Micro-Computed Tomography Assessment of the Quality of Obturation (Voids) of Single-Canal Maxillary Second Premolars by the Lateral Compaction versus Continuous Warm Vertical Condensation Techniques

**DOI:** 10.30476/dentjods.2024.99581.2159

**Published:** 2025-03-01

**Authors:** Romina Hajipour, Maryam Zare Jahromi, Masood Khabiri

**Affiliations:** 1 Dental Research Center, Research Institute of Dental Sciences, Dental School, Shahid Beheshti University of Medical Sciences, Tehran, Iran; 2 Dept. of Endodontics, Faculty of Dentistry, Isfahan (Khorasgan) Branch, Islamic Azad University, Isfahan, Iran

**Keywords:** Root Canal Therapy, X-Ray Microtomography, Root Canal Obturation

## Abstract

**Statement of the Problem:**

One of the most important stages of root canal treatment is obturation for the root canal, an obturation with less voids will have fewer treatment complications in the future.

**Purpose:**

This study was conducted to compare the quality of obturation of single-canal maxillary second premolars by the cold lateral compaction (CLC) versus continuous warm vertical condensation (CWVC) techniques using micro-CT.

**Materials and Method:**

In this experimental study, 36 extracted single-canal maxillary premolars were selected. The root canals were instrumented by Denco Blue rotary files. The teeth were randomly assigned to three groups (n=12) of control (no root filling), root canal obturation with CLC technique, and root canal obturation with CWVC technique. Next, they underwent micro-CT, and the mean volume and volume percentage (VP) of voids were calculated in the apical, middle, and coronal thirds of the root canals. Data were analyzed using one-way ANOVA, the Kruskal-Wallis, Mann-Whitney U, Bonferroni, Dunnett, Tukey, and independent t-tests (*p* Value<0.05).

**Results:**

In the coronal third, no significant difference was found between the CLC and CWVC groups in the mean volume of voids (*p*= 0.273), the mean volume of filled space (*p*= 0.419), or the VP of voids (*p*= 0.605). The highest mean volume and VP of voids were recorded in the coronal part of the group CWVC (*p*> 0.05). The lowest mean volume and VP of voids was recorded in the apical third in CWVC group (*p*< 0.05).

**Conclusion:**

None of the obturation techniques could provide a void-free root filling. Two techniques showed no significant difference regarding the mean volume and VP of voids in obturation of single-canal maxillary second premolars.

## Introduction

The success of endodontic treatment depends on three main steps of effective debridement, efficient disinfection, and optimal obturation [ 1 - 2 ]. Ideally, the root filling material should three-dimensionally and homogenously fill the entire root canal space; however, this goal cannot be perfectly achieved in the clinical setting [ 3 ]. Resultantly, the microorganisms lodged in accessory canals or the periapical region may proliferate and colonize the residual voids [ 3 ]. 

The cold lateral compaction (CLC) technique is the widely practiced method of root canal obturation [ 4 ]. The CLC technique does not have high technical sensitivity, and is affordable. Nonetheless, the auxiliary (lateral) gutta-percha points used in this technique cannot ideally adapt to the root canal wall irregularities and some voids may remain [ 5 ]. 

The continuous warm vertical condensation (CWVC) technique is another root canal obturation technique, which was introduced to improve homogeneity and adaptation of root filling material to the root canals. In this technique, the master gutta-percha point along with sealer is introduced into the canal to the working length; the gutta-percha point is then packed into the canal by an element to 5 mm to the apex. Next, the residual space is filled with injectable thermoplastic gutta-percha [ 6 - 7 ]. 

Micro-computed tomography (micro-CT) is a novel highly accurate technique for reproducible assessment of specimens without their destruction [ 8 - 11 ]. Micro-CT was capable of precise 3D reconstruction of root canal filling [ 12 ]. Micro-CT was developed in early 1980, as a non-invasive and non-destructive imaging modality to obtain 3D images. It operates based on convergence of X-ray beams on the specimen and their receipt by a sensor. The irradiated X-ray is then converted to digital images [ 13 ]. 

In endodontic treatment, selection of an optimal root canal obturation technique is imperative to prevent root canal reinfection. 

Considering all the above, the purpose of this study was to compare the quality of obturation of single-canal maxillary second premolars by the CLC versus CWVC techniques using micro-CT. 

## Materials and Method

This *in vitro*, experimental study was conducted on 36 single-canal maxillary second premolars extracted for reasons not related to this study such as orthodontic treatment or poor periodontal prognosis. The study was approved by the Ethics Committee of School of Dentistry, Islamic Azad University, Khorasgan Branch (IR.IAU.KHUISF.REC.1399.062). 

### Eligibility criteria

Mature teeth with closed apex, without root resorption, absence of tooth anomalies or calcification or anatomical variations or previous endodontic treatment were included in the study. 

Schneider's method was used to measure the curve canals and teeth with a curve of 15 to 20 degrees were selected [ 14 ].

Each tooth underwent digital periapical radiography in labial and proximal directions to ensure meeting the eligibility criteria.

### Sample size

The minimum sample size was calculated by random and simple method to be 12 in each of the three groups assuming alpha=0.5, beta=0.2, study power of 80%, mean values of 1.02 and 4.1 in the two groups, and standard deviations of 0.42 and 2.7 in the two groups. To increase accuracy, 12 teeth were included in each group. 

### Specimen preparation

The teeth were cleaned with 5.25% sodium hypochlorite (Morvabon, Iran) after extraction and stored in saline until use. Eligible teeth were then selected and decoronated with a diamond fissure bur (Teezkavan, Iran) under water coolant such that 14mm of root length remained for the purpose of standardization. 

### Root canal instrumentation

A K-file #10 (Mani, Japan) was introduced into the canal until its tip was visible at the apex; 1mm was subtracted from this length to determine the working length. After creating a glide path with a K-file#15 and radiographic confirmation of the working length, teeth with initial files larger than #30 were excluded. Denco Blue rotary files (Denco, China) up to F3 were used for root canal shaping with 2 N/cm torque and 250 rpm speed as instructed by the manufacturer with an endomotor (NSK, Japan). In the process of root canal cleaning and shaping, 5.25% sodium hypochlorite was frequently used. In addition, the canal path was preserved and the patency was verified by using a K-file#10. The root canals were then rinsed with saline, followed by 17% EDTA (Meta BioMed, Chungju, Korea) for 1 minute, and a final rinse with saline was then performed. 

### Root canal obturation

The teeth were randomly assigned to three groups (n=12) as follows:

### Negative control group

The root canals were not obturated in this group.

### CLC group

This group underwent obturation with the CLC technique using gutta-percha (DiaFil TM, DiaDent, Korea) and AH-Plus sealer (Dentsply Maillefer, Ballaigues, Switzerland). A #30/2% tapered gutta-percha point with tug-back was selected as the master cone. If the tug back was not achieved or the #30/2% gutta percha did not have a stop, the tooth was excluded and replaced by another one. After radiographic confirmation of the optimal length of the master cone, the root canals were dried with paper points (Dentsply Maillefer, Ballaigues, Switzerland). The master cone was dipped in sealer and inserted into the canal to the working length. A spreader (Mani, Japan), reaching 2-3mm of the working length [ 15 ], was used and accessory gutta-percha points (one size smaller than the spreader) were used to fill the canal until the spreader could not penetrate into the canal by more than 2mm. 

Excess gutta-percha was removed by an excavator, and a cold plugger was used to pack the root filling at the orifice for 5 seconds. Finally, the orifice was sealed with auto-polymerizing glass ionomer cement (GC, Japan). 

### CWVC group

ProTaper F3 gutta-percha (Dentsply Maillefer, Ballaigues, Switzerland) along with AH Plus resin sealer was placed in the canal, and its proper length was radiographically confirmed. It was cut 3-4mm above the working length by a plugger (Meta BioMed, Chungju, Korea) heated to 200°C to create an apical plug. Next, a radiographic image was obtained to ensure optimal quality of the apical plug. The sealer was applied again in the residual root canal space, and thermoplasticized gutta-percha was injected into the canal to fill the canal completely (FastPack; Eighteeth, China). Finally, the canal orifice was sealed with glass ionomer cement. After obturation, the teeth were incubated at 37° and 100% humidity to allow sealer setting. 

All teeth kept for 24 hours in the incubator with 100% humidity and temperature of 37℃. All this procedure was carried out by a single experienced operator.

### Micro-CT

**Figure 1:**
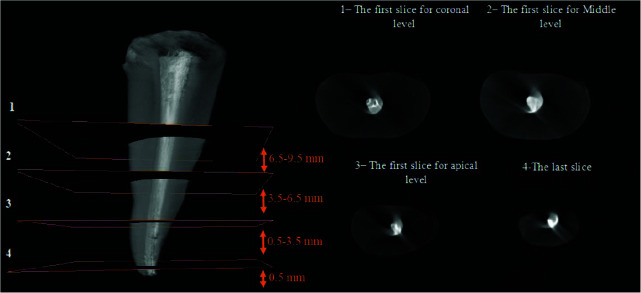
Micro-CT scan of the entire canal along with middle and apical third axial coronal sections in the cold lateral compaction (CLC) group

**Figure 2:**
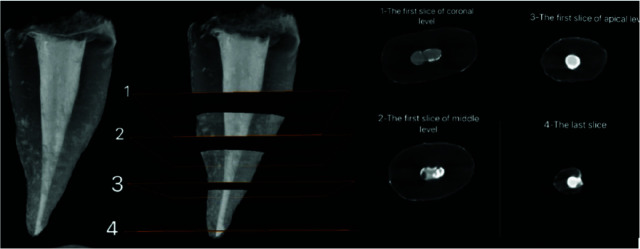
Micro-CT scan of the entire canal along with middle and apical third axial coronal sections in continuous warm vertical condensation (CWVC) group

**Figure 3:**
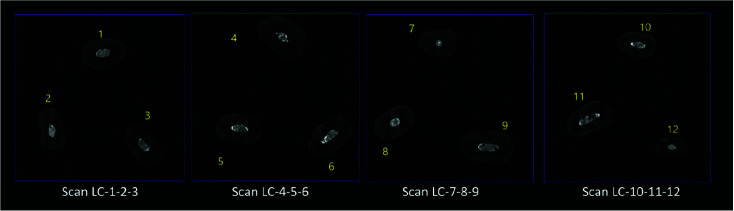
Three-dimensional micro-CT scan of specimens in the cold lateral compaction (CLC) group

**Figure 4:**
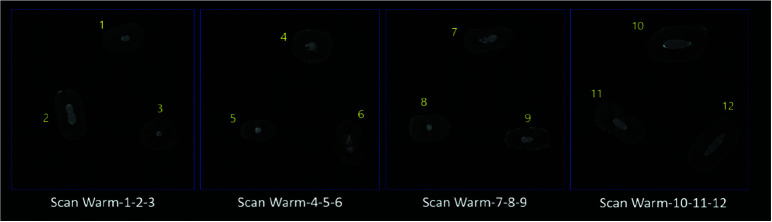
Three-dimensional micro-CT scan of specimens in the continuous warm vertical condensation (CWVC) group

The roots were scanned with a micro-CT scanner (Lotus, Behin Negareh, Iran) with 15µm pixel size, 80 kV voltage, 100 µA amperage, and 45 minutes time (per each root). To reconstruct 2D images, cross-sectional slices were obtained in the axial plane with 45-µm slice thickness perpendicular from the root apex towards the coronal region. The 2D images were reconstructed using Lotus *in vivo* ACQ software (Figures 1 to 4). Negative group was used for the calibration of the micro-CT scanner. Empty spaces (voids) and filled areas were distinguished according to their density on 2D images, and were then reconstructed three-dimensionally using Lotus *in vivo* REC software; subsequently, their volume was calculated. The volume percentage (VP) of voids was calculated by dividing the volume of voids by the total volume of the root filling multiplied by 100, and reported separately in the different parts, and also the entire root length [ 14 ]. 

### Statistical analysis

Data were analyzed by SPSS version 20 (SPSS Inc., IL, USA). Accordingly, comparisons were made by one-way ANOVA, the Kruskal-Wallis, Mann-Whitney U, Bonferroni, Dunnett, Tukey, and independent t-tests at 0.05 level of significance. 

## Results

Table 1 presents the mean volume of voids and filled spaces, and the VP of voids in the CLC and WVC groups in the apical, middle, and coronal thirds. 

**Table 1:** Mean volume of voids and filled spaces, and the volume percentage (VP) of voids in the CLC and CWVC groups in the apical, middle, and coronal thirds

		Volume of voids		Volume of filled areas		VP	
		Mean	Std. deviation	Mean	Std. deviation	Mean	Std. deviation
CLC	Apical	0.05304	0.040	1.1966	0.367	4.349	2.723
	Middle	0.0789	0.0720	2.1652	0.692	3.4354	2.449
	Coronal	0.1292	0.108	3.7069	1.349	3.3309	2.167
	Entire canal	0.2614	0.210	7.0688	2.312	3.5569	2.169
CWVC	Apical	0.0132	.009	1.1024	0.262	1.1792	0.808
	Middle	0.1544	0.132	2.5088	0.799	6.4839	7.431
	Coronal	0.1659	0.099	4.5344	1.967	3.8377	2.544
	Entire canal	0.3335	0.203	8.1458	2.860	4.3031	3.534

CLC: Cold lateral compaction; CWVC: Continuous warm vertical condensation; VP: Volume percentage

Comparison of CLC and CWVC techniques regarding the mean volume of voids and filled areas and VP of voids separately in the apical, middle, and coronal thirds are shown in Table 2.

**Table 2:** Comparison of the mean volume of voids and filled canal space and volume percentage (VP) of voids between the CLC and WVC groups in the apical, middle and coronal thirds

		Volume of voids		Volume of filled areas		VP	
		Mean	Std. deviation	Mean	Std. deviation	Mean	Std. deviation
Apical third	CLC	0.05304	0.040	1.1966	0.367	4.349	2.723
	CWVC	0.0132	0.009	1.1024	0.262	1.1792	0.808
	Statistic	3.28		0.722		-3.233	
	*p* Value	0.006		0.478		0.001	
Middle third	CLC	.0789	.0720	2.1652	.692	3.4354	2.449
	WVC	.1544	.132	2.5088	0.799	6.4839	7.431
	Statistic	-1.386		-1.328		-1.155	
	*p* Value	0.166		0.184		0.248	
Coronal third	CLC	.1292	0.108	3.7069	1.349	3.3309	2.167
	CWVC	.1659	0.099	4.5344	1.967	3.8377	2.544
	Statistic	-1.097		-0.808		-0.525	
	*p* Value	0.273		0.419		0.605	

CLC: Cold lateral compaction; WVC: Warm vertical condensation

In the apical third, the mean volume of voids in the CLC group was significantly higher than that in the CWVC group (*p*= 0.006). The VP of voids in the CLC group was also significantly higher than that in the CWVC group (*p*= 0.001). The difference in the mean volume of filled space was not significant between the two groups (*p*= 0.478).

In the middle third, no significant difference was found between the CLC and CWVC groups in the mean volume of voids (*p*= 0.166), mean volume of filled space (*p*= 0.184), or the VP of voids (*p*= 0.248). 

In the coronal third, no significant difference was found between the CLC and CWVC groups in the mean volume of voids (*p*= 0.273), mean volume of filled space (*p*= 0.419), or the VP of voids (*p*= 0.605). 

Concerning the mean volume of voids and filled are-as and VP of voids in the entire root canal length, no significant difference was found between the CLC and CWVC groups in the mean volume of voids (*p*= 0.299), mean volume of filled space (*p*= 0.443), or the VP of voids (*p*= 0.755) in the entire root canal length (Table 3). 

**Table 3:** Comparison of CLC and WVC techniques regarding the mean volume of voids and filled areas and volume percentage (VP) of voids in the entire root canal length

	Volume of voids		Volume of filled areas		VP	
	Mean	Std. deviation	Mean	Std. deviation	Mean	Std. deviation
CLC	.2614	.210	7.0688	2.312	3.5569	2.169
CWVC	.3335	.203	8.1458	2.860	4.3031	3.534
Statistic	-1.039		-0.808		-0.346	
*p* Value	0.299		0.443		0.755	

CLC: Cold lateral compaction; WVC: Warm vertical condensation

## Discussion

This study compared the percentage of voids of single-canal maxillary second premolars by the CLC versus CWVC techniques using micro-CT. the mean volume and VP of voids in the root canals of maxillary second premolars were not significantly different between the CLC and WVC groups in this study. The lowest volume of voids was recorded in the apical third in the WVC group, which was significantly lower than that in the apical third in the CLC group. In addition, the mean volume and VP of voids in the apical third was significantly lower than the corresponding values in the middle and coronal thirds in the WVC group.

Several methods have been proposed for assessment of the quality of root canal obturation, such as the bacterial leakage model, dye penetration technique, fluid filtration technique, confocal laser scanning microscopy, radioisotope tracing, scanning electron microscopy, and cone-beam computed tomography.

Micro-CT is a novel highly accurate technique for reproducible assessment of specimens without their destruction [ 8 - 11 ]. A previous study compared micro-CT and histological analysis and revealed that micro-CT was capable of precise 3D reconstruction of root canal filling [ 12 ]. Micro-CT was developed in early 1980, as a non-invasive and non-destructive imaging modality to obtain 3D images. It operates based on convergence of X-ray beams on the specimen and their receipt by a sensor. The irradiated X-ray is then converted to digital images [ 13 ].

Keleş *et al.* [ 16 ] evaluated the obturation quality of oval-shaped canals filled by the CLC and WVC techniques using micro-CT. They found that the volume of voids was significantly higher in CLC group; however, the difference in this regard was not significant between the two groups in the apical third [ 16 ]. Their results were different from the present findings since in the present study, the mean volume and VP of voids were not significantly different between the two groups, but this difference was significant in the apical third. The difference between the two studies may be due to evaluation of different tooth types, and experience and expertise of the operators. Almohaimede *et al.* [ 17 ] compared the filling porosity of two different obturation techniques in 40 single-canal teeth using micro-CT. They compared CLC and continuous-wave techniques with gutta and AH Plus, and found that the volume of voids in the different thirds in the continuous-wave group was higher than that in the CLC group. The highest filling porosity was noted in the apical third in continuous-wave group but no statistically significant difference was found between different obturation techniques. Their results were similar to our study, with the difference that the lowest mean volume and VP of voids in the present study was recorded in the apical third of WVC group, and the two groups were not significantly different in this regard in other parts of the root. Amida *et al.* [ 18 ] compared the obturation sealability of CLC, GuttaCore®, and WVC technique using micro-CT. They used extracted maxillary central incisors and found the highest volume of voids in the apical third in all techniques. Although the GuttaCore® group showed fewer voids than the other two techniques, they all had similar sealing ability, and the GuttaCore® and CLC groups had no significant difference in the mean volume and VP of voids [ 18 ]. Their results, regarding no significant difference between the CLC and CWVC groups, were in agreement with the present findings. However, they reported the highest volume of voids in the apical third in all groups while the lowest volume and VP of voids were found in the apical third of CWVC group in the present study. 

Both obturation techniques and both sealers produced voids, which is in accordance with the findings of Celikten *et al.* [ 19 ], who suggested that voids are mainly correlated with the root canal anatomy rather than the root canal filling material or technique.

The round shape of the canal may limit the penetration of the spreader in the apical part in the lateral compaction technique and prevent the insertion of the lateral gutta to the length of working. This problem does not exist in oval canals [ 20 ]. For this reason, maxillary premolar teeth, which have an oval canal, were selected for this study.

The VP of voids is commonly calculated to assess the quality of root filling [ 14 , 21 ] because bacteria and their byproducts can colonize the voids and compromise the success of the treatment. Thus, optimal seal in the entire root length is required [ 14 ]. The significantly lower mean volume and VP of voids in the apical part in WVC group may suggest the superiority of this technique to CLC in possible prevention of leakage of microorganisms in infected canals.

 Considering the importance of cleaning, shaping, and appropriate obturation of the apical part to maintain seal and prevent re-contamination and presence of lateral canals, this area affects the treatment results; therefore, using the obturation method with the least amount of voids in this area is important [ 1 , 4 , 22 ].

Considering the lowest amount of voids in the apical part of the CWVC in our study, this technique will be important and effective.

The *in vitro* design of the study can be considered as a limitation, which confines the generalization of results to the clinical setting. Future studies are required to compare the results of micro-CT with the results of sealer dissolution and leakage in the long-term to find more reliable information regarding the long-term sealing ability of sealers and also about root fillings in different obturation techniques. Moreover, similar studies are required using silicone sealers in comparison with resin and calcium silicate-based sealers, and also different teeth with variable levels of root curvature. Crack formation should also be compared following root canal obturation with different techniques. Cone-beam computed tomography might be used for further investigations in this regard.

## Conclusion

None of the tested obturation techniques could provide a void-free root filling. The CLC and CWVC techniques showed no significant difference regarding the mean and VP of voids in obturation of single-canal maxillary second premolars. The apical third in the CWVC group showed the lowest mean percentage of voids, which was significantly lower than that in the apical third of the CLC group.
